# Impact of Fulvic Acid and *Acidithiobacillus ferrooxidan* Inoculum Amount on the Formation of Secondary Iron Minerals

**DOI:** 10.3390/ijerph20064736

**Published:** 2023-03-08

**Authors:** Haitao Huang, Kanghui Geng, Chong Wang, Xianhui Wu, Caichun Wei

**Affiliations:** 1 College of Environmental Science and Engineering, Guilin University of Technology, Guilin 541004, China; hthuang@126.com (H.H.);; 2 Guangxi Key Laboratory of Environmental Pollution Control Theory and Technology, Guilin University of Technology, Guilin 541004, China; 3 Guangxi Collaborative Innovation Center for Water Pollution Control and Water Safety in Karst Areas, Guilin 541004, China; 4 Guangxi Modern Industry College of Ecology and Environmental Protection, Guilin University of Technology, Guilin 541004, China

**Keywords:** acid mine drainage, fulvic acid, *Acidithiobacillus ferrooxidan*, schwertmannite

## Abstract

The catalytic oxidation of Fe^2+^ by *Acidithiobacillus ferrooxidan* (*A. ferrooxidans*) and the synthesis of iron sulfate-based secondary minerals is considered to be of great significance to the treatment of acid mine drainage (AMD). Along these lines, in this work, the shaker experiment was carried out to study the underlying mechanism of the inoculation amount of fulvic acid (FA) and *A. ferrooxidans* on the synthesis process of secondary minerals. From the acquired results, it was demonstrated that the oxidation rate of Fe^2+^ increased with the increase in the concentration of fulvic acid in the range of 0.1–0.2 g/L. On top of that, the concentration of fulvic acid in the range of 0.3–0.5 g/L inhibited the activity of *A. ferrooxidans*. However, *A. ferrooxidans* retained its activity, and the complete oxidation time of Fe^2+^ was delayed. When the concentration of fulvic acid was 0.3 g/L, the TFe (total iron) precipitation efficiency was 30.2%. Interestingly, when 0.2 g/L fulvic acid was added to different inoculum systems, the incorporation of a higher inoculum amount of *A. ferrooxidans* led to an increased oxidation rate. On the contrary, the lower inoculum amount yielded a more obvious effect of the fulvic acid. From the mineralogical characteristics, it was also revealed that a fulvic acid concentration of 0.2 g/L and different inoculation amounts of *A. ferrooxidans* did not change the mineral facies, whereas pure schwertmannite was obtained.

## 1. Introduction

Acid mine drainage (AMD) is usually produced by biochemical reactions during or after the mining process. It is characterized by the presence of large amounts of Fe and S elements, a low pH ranging from about 2 to 3.5, and an excessive heavy metal (metalloid) content [1], which can severely pollute the soil and water environment if discharged randomly without treatment. The current methods of treating AMD include acid-base neutralization, coagulation sedimentation, chemical oxidation, constructed wetland, and biological methods [2]. The neutralization method is widely used in AMD treatment [3,4], with a usage rate of over 90% [5]. The precipitation efficiency of Fe^2+^ in AMD is higher at a pH = 8–9 than at a pH ≈ 3 [6], while oxidative pretreatment is often used to treat wastewater and *A. ferrooxidans* can play such a role. *A. ferrooxidans* are chemoautotrophic, exclusively aerobic, with CO_2_ as their carbon source, inorganic nitrogen-containing compounds as nitrogen sources. Moreover, *A. ferrooxidans* use Fe^2+^ and reduced sulfur compounds as energy substances. They are mainly present in environments with pH values from 1 to 3 [7,8], in various successful applications in hydrometallurgy [9], during the coal and flue gas desulfurization procedures [10,11,12], acid wastewater treatment [13], sludge treatment [14,15], etc.

More specifically, during the AMD process, *A. ferrooxidans* play an important role in the formation of secondary minerals. Fe^2+^ is first oxidized to Fe^3+^ and then hydrolyzed into secondary minerals, such as jarosite (K, Na, NH_4_, H_3_O)Fe_3_(SO_4_)_2_(OH)_6_ and schwertmannite Fe_8_O_8_(OH)_6_(SO_4_)_4_. This process is of great significance for removing Fe^2+^, Fe^3+^, and SO_4_^2−^ in the solution, and also plays a role in the adsorption or co-precipitation of heavy metals (metalloid) in the solution [16]. For example, schwertmannite possesses hydroxyl groups, sulfate groups, and a large specific surface area. It has been reported in the literature that the adsorption capacity of As by schwertmannite has a good adsorption effect on both As and Cr [17,18]. Cu^2+^ and Pb^2+^ also exist in the form of ternary complexes on the surface of schwertmannite [19], which plays an important role in the migration of heavy metals and has a potential application value.

Humic substances exist in large quantities in nature and contain various functional groups, such as carboxyl, hydroxyl, quinone, and other functional groups [20]. These functional groups can complex or chelate heavy metals (metalloid) in the solution or soil and can also affect the migration of heavy metals [21]. Interestingly, in aquatic systems, fulvic acid (FA) and humic acid account for about 30–35% of organic matter [22], but little attention is usually paid to the impact of these substances on wastewater treatment. Fulvic acid is a highly oxidized, biologically stable, and water-soluble natural complexing agent [23]. In addition, fulvic acid has a wide range of pH dissolution, is soluble in acid and alkali, and contains carboxyl groups, similarly to general organic acids (e.g., formic acid, acetic acid, and propionic acid). It also consists of active functional groups such as quinones that can accelerate electron transfer [24]. The average molecular weight of fulvic acid is lower than that of humic acid. It can bind minerals and elements to its molecular structure [25]. Fulvic acid can combine with chromium, copper, and nickel in water and be used as a potential toxic metal reducing agent in water [26]. Coal-based fulvic acid was characterized and the molecular model of fulvic acid has been constructed [27]. However, the impact of fulvic acid on Fe^2+^ oxidation by *A. ferrooxidans* in AMD has been scarcely reported in the literature. Under this direction, in this experiment fulvic acid was used as the research object to explore the influence of fulvic acid concentration on the activity of *A. ferrooxidans*, as well as the impact of the different inoculation amount on the formation of secondary minerals in the presence of fulvic acid. The main goal of this work is to provide a solid theoretical basis for environmental management applications.

## 2. Materials and Methods

### 2.1. Experimental Material

The employed fulvic acid (Hefei BASF Biotechnology Co., Ltd., Hefei, China) was analytically pure at 98%. *A. ferrooxidans* (ATCC23270) was cultured in 9K medium with the following configuration [28]: all reagents were analytically pure, (NH_4_)_2_SO_4_ 60.0 g, KCl 2 g, K_2_HPO_4_ 10.0 g, Ca(NO_3_)_2_∙4H_2_O 0.2 g, and MgSO_4_∙7H_2_O 10.0 g (All reagents from Xilong Science) and were dissolved in 1 L of deionized water, with 1:1 H_2_SO_4_ to adjust the pH value to 2.5 until the solution became clear. For the preparation of *A. ferrooxidans* cell suspensions, the following steps were applied: 12.5 mL of 9K medium was added to a 500 mL conical flask, and *A. ferrooxidans* (50 mL bacterial solution) were inoculated in a 9K medium. Finally, the volume of the solution in the conical flask was 250 mL, the pH was adjusted to 2.5 (±0.2), the rotation speed was 180 rpm, and the temperature was 28 °C. The culture was stopped at the late exponential growth stage (about 2–3 d). Subsequently, the culture was filtered to remove the precipitate, and the filtrate was centrifuged at 10,000× *g* (4 °C for 10 min) to collect the bacteria. Then, the bacteria were washed three times with acid water (H_2_SO_4_) with pH = 1.5, to fully remove the hetero-ion. Original bacterial solution was suspended with acid water (H_2_SO_4_) with pH = 2.5. The cell density was about 4 × 10^8^ cells/mL. A total of 50 mL of the original bacterial solution was concentrated into 1 mL of (20% *v*/*v*) bacterial solution. A total of 2.5 g of fulvic acid was fixed in a 250 mL volumetric flask to yield a concentration of 10 mg/mL of fulvic acid solution.

### 2.2. Experimental Setup

Experiment 1: In a series of 500 mL conical flasks, five groups of experiments were set up, with three parallel samples in each set. Particularly, 11.06 g of H_2_SO_4_-7H_2_O was accurately weighed in each flask to derive a concentration of 160 mmoL/L of Fe^2+^ and dissolved in deionized water. The concentration of fulvic acid was 0.1, 0.2, 0.3, 0.4, and 0.5 g/L, respectively. The pH value of the system was adjusted to 2.5 (±0.2) with 1 mol/L sulfuric acid. Then, 1 mL of the bacterial suspension that was prepared in advance was added (20% *v*/*v*) and the final volume of the solution was 250 mL.

Experiment 2: Setting up 6 groups of experiments, where each group of experiments set up 3 parallel samples. Initially, 11.06 g of H_2_SO_4_-7H_2_O was accurately weighed in each conical flask to attain a Fe^2+^ concentration of 160 mmoL/L (8.96 g/L). Next, a certain amount of Ca^2+^ was added to the flasks to make the concentration of Ca^2+^ in each system 0.3 g/L. The inoculation amounts were 5% (1 × 10^8^ cells/mL), 10 % (2 × 10^8^ cells/mL), and 20% (4 × 10^8^ cells/mL), while a certain amount of fulvic acid was added to the experimental group with the same inoculation amount to obtain a concentration of 0.2 g/L for the fulvic acid. The pH of the system was adjusted to 2.5 (±0.2) with 1 mol/L sulfuric acid, and a final solution volume of 250 mL was derived.

The above-mentioned conical flasks were placed in a constant temperature shaker with a temperature of 28 °C and a rotation speed of 180 rpm. The samples were taken at regular intervals to determine the pH, Fe^2+^, and Fe^3+^ concentrations in the solution at the following times: 12, 24, 36, 48, 72, 96, 120, and 144 h. Additionally, at the end of the experiment, the precipitates were filtered through qualitative filter paper. First, the precipitates were washed with pH = 1.5 sulfuric acid to remove soluble ions and then were rinsed with deionized water to become neutralized. After drying at 60 °C for 24 h, they were weighed and sieved.

### 2.3. Determination Method and Data Analysis

The liquid samples were collected using a 0.22 μm filter syringe, while the Fe^2+^ and Fe^3+^ were determined by conducting phenanthroline colorimetry measurements. The pH value of the solution was determined by using a pH meter (using pHS-3C pH meter, Shanghai LeiMag). The mineral phase was determined by using an X-ray diffractometer (X’Pert 3 Powder, Panaco, The Netherlands). The internal groups of minerals were determined by utilizing a Fourier transform infrared spectrometer (Frontier FT-IR). The acquired experimental data were analyzed using the Origin mapping software.

## 3. Results and Discussion

### 3.1. Impact of Fulvic Acid on the Fe^2+^ Oxidation Efficiency and Rate of Secondary Mineral Synthesis Systems

**Figure 1:**
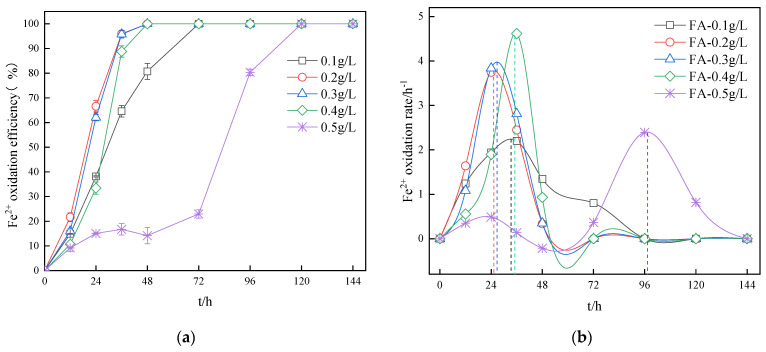
Impact of FA on the Fe^2+^ oxidation efficiency (**a**) and rate (**b**) in systems.

The effective oxidation of Fe^2+^ in the AMD environment is directly related to the precipitation of Fe^3+^ in the system, which in turn affects the synthesis of the secondary minerals. The influence of the fulvic acid concentration on the oxidation efficiency and rate of Fe^2+^ in the biosynthetic secondary mineral system is depicted in Figure 1. It can be seen from Figure 1a, that the different concentrations of fulvic acid had a significant impact on the oxidation efficiency of Fe^2+^. After 12 h, the Fe^2+^ oxidation efficiency of the FA—0.1 g/L system was lower than that of both FA—0.2 g/L and FA—0.3 g/L, and was higher than that of FA—0.4 g/L and FA—0.5 g/L. Fulvic acid as an organic acid inhibited the oxidation efficiency of *A. ferrooxidans*. It has been reported in the literature that organic matter and small molecular organic acids (formic acid, acetic acid, etc.) can be toxic to *A. ferrooxidans* [29]. The underlying reason for this effect is the action of carboxyl groups on the bacterial cell membrane, which causes permeability changes, as well as the presence of quinone groups in fulvic acid, which facilitates electron transfer [30]. In the FA—0.4 g/L system, the oxidation efficiency of Fe^2+^ was lower than that of the FA—0.1 g/L system before 24 h, and vice versa after 24 h. The inhibitory effect of fulvic acid in the FA—0.5 g/L system lasted for 72 h. When the electron transfer effect was relatively stronger than the toxic effect of the fulvic acid carboxyl group, the biological oxidation of Fe^2+^ could be promoted. According to Figure 1b, the highest oxidation rate was reached first at a concentration of 0.2 g/L fulvic acid, and the highest oxidation rate was delayed when the fulvic acid concentration was lower or higher than 0.2 g/L. The oxidation rate was the highest at 36 h when the fulvic acid concentration was 0.4 g/L. It can be seen that when the concentration of the fulvic acid is lower than the value of 0.2 g/L, the low oxidation rate was due to the low concentration of fulvic acid. The increase in the oxidation rate when the fulvic acid concentration was high was caused by the increase in the quinone content of the system. As far as the delay of the maximum oxidation rate is concerned, it was caused by the inhibition of the carboxyl group in fulvic acid on *A. ferrooxidans*.

### 3.2. Impact of Fulvic Acid on the TFe Precipitation Efficiency in Secondary Mineral Synthesis Systems

**Figure 2:**
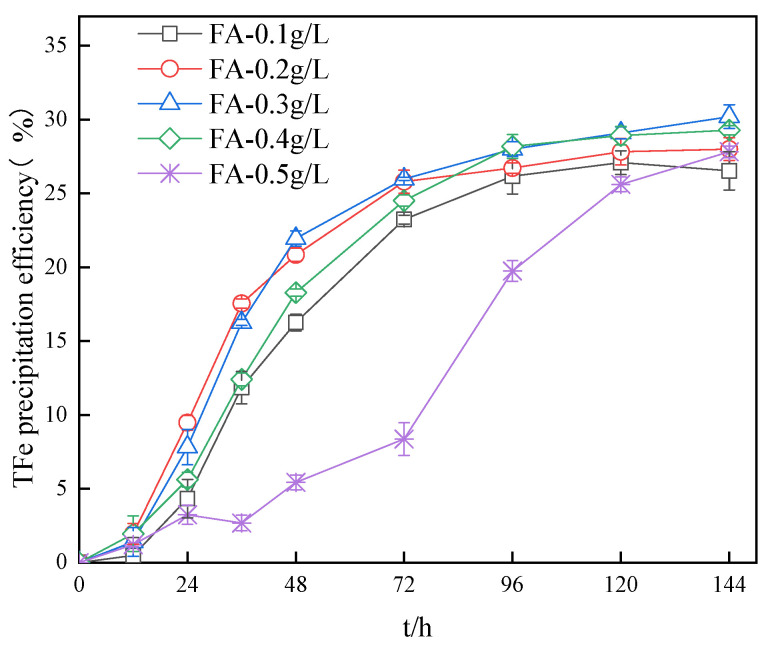
Impact of FA on the TFe precipitation efficiency in systems.

The impact of the different concentrations of fulvic acid on the precipitation efficiency of TFe in solution is displayed in Figure 2. The Fe^3+^ supply rate plays an important role in the TFe precipitation, and the TFe precipitation efficiency increased with the rise of the Fe^2+^ oxidation efficiency before 36 h. Figure 1a shows that for systems with fulvic acid concentrations of 0.1 to 0.4 g/L, Fe^2+^ was completely oxidized after 72 h, and the experiment was completed after 144 h. The final TFe precipitation efficiency was 26.5%, 28.0%, 30.2%, and 29.28%, respectively. The increase in the fulvic acid concentration can promote the hydrolysis and precipitation of Fe^3+^ to some extent. It has been reported in the literature that Mg2Al layered double hydroxides (LDH) and fulvic acid (FA) can be combined to form nuclei [31]. The low concentration of fulvic acid in this experiment may have similar properties to the hydroxides produced in the solution. Furthermore, the low concentrations of tryptophan could favor crystal nucleation, which in turn promotes the formation of secondary minerals. More specifically, when there is an excess of tryptophan, tryptophan is adsorbed to the surface of secondary minerals and inhibits their formation. It should also be stressed that the TFe precipitation efficiency of the 0.5 g/L fulvic acid system at 144 h was 27.81%. Although it was not the highest, the complete oxidation time of Fe^2+^ in this system was 120 h. From Figure 2, a significant increasing trend can still be recorded, which remains to be studied to determine whether fulvic acid is excessive at this concentration.

### 3.3. Impact of A. ferrooxidans Inoculum Amount on the pH in the Secondary Mineral Synthesis System

**Figure 3:**
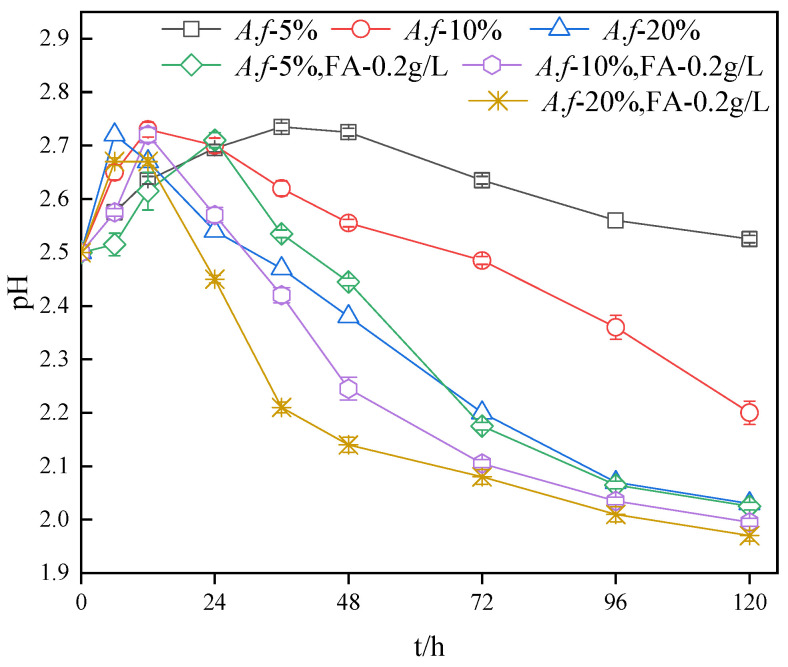
Impact of *A. ferrooxidans* inoculation amount on the pH in systems.

The pH change was thoroughly investigated under the experimental conditions of pH = 2.5 and Fe^2+^ concentration of 8.96 g/L. As can be observed from Figure 3, all the pH changes showed a trend of increasing first and then decreasing. More specifically, for the two processes of Fe^2+^ oxidation acid consumption and Fe^3+^ hydrolysis acid production, the following set of equations apply [32]:

(1) $$4{\mathrm{Fe}}^{2+}+O_{2}+4H^{+}\overset{A.\;ferrooxidans}{\to }4{\mathrm{Fe}}^{3+}+2H_{2}O$$

8Fe^3+^ + 14H_2_O + SO_4_^2−^ = Fe_8_O_8_(OH)_6_SO_4_ + 22H^+^ (2)

After 6 h, a higher inoculation amount led to a faster pH rise. The increase in the inoculation amount promoted the oxidation of Fe^2+^ and accelerated the consumption of H^+^. The pH of the fulvic acid group was lower than that of the equal bacterial inoculation amount. Fulvic acid has an inhibitory effect on the bacteria in two aspects, which is detrimental to the consumption of H^+^ in the solution. Second, the chelation and complexation of fulvic acid with Fe^2+^ will release a certain amount of H^+^, resulting in a relatively slow rise in the pH value. As the pH of each system decreased, the pH of the experimental system with the higher bacterial inoculum decreased more rapidly. For example, after 48 h without fulvic acid (the annotation in Figure 3 is written as FA), the pH of *A. ferrooxidans* (the annotation in Figure 3 is written as *A. f.*) inoculated at 5%, 10%, and 20% was 2.73, 2.56, and 2.38, respectively. In addition, the pH of the system with fulvic acid decreased more rapidly than that of the system with an equal bacterial inoculation amount. For example, after 36 h, the pH values of the *A.f*—20% + FA—0.2g/L and *A.f*—20% systems were 2.21 and 2.47, respectively, with a decrease of 0.26 in the former compared to the latter. This result indicates that both the addition of fulvic acid and the increase in the inoculum level of *A. ferrooxidans* can indirectly contribute to the decrease in the pH value.

### 3.4. Impact of A. ferrooxidans Inoculum Amount on the Fe^2+^ Oxidation Efficiency in Secondary Mineral Synthesis Systems

**Figure 4:**
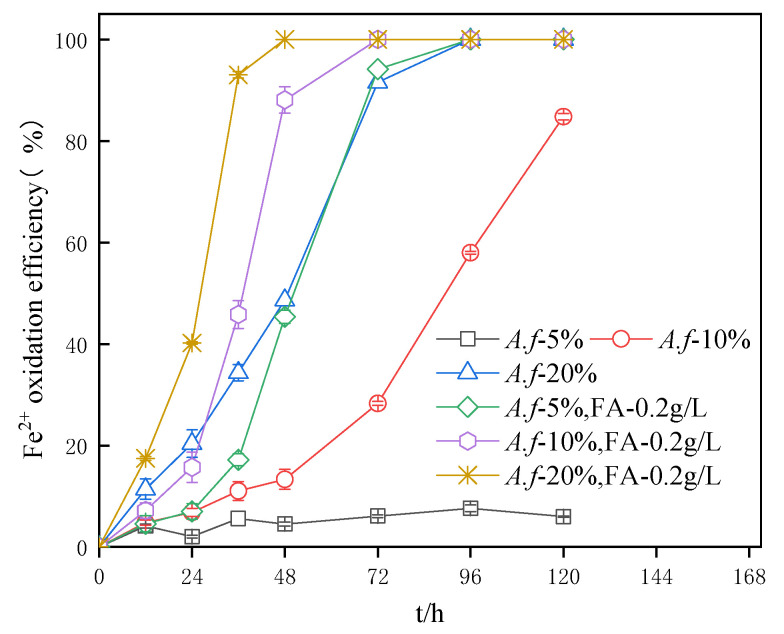
Impact of *A. ferrooxidans* inoculation amount on the Fe^2+^ oxidation efficiency in systems.

According to Figure 4, regardless of whether fulvic acid was added or not, the existence of a higher inoculum of *A. ferrooxidans* in the system led to an increased oxidation efficiency of Fe^2+^ at the same time. For example, the oxidation efficiencies of Fe^2+^ in the system with 5%, 10%, and 2% inoculation amounts after 96 h were 5%, 58%, and 100%, respectively. The oxidation efficiency of Fe^2+^ in the system with fulvic acid was also higher than that in the system that was inoculated with an equal amount of bacteria. We have to underline that the addition of fulvic acid can adapt to all bacterial inoculation systems. It is also worth noting that the oxidation efficiency of Fe^2+^ in the system with 5% inoculation still did not reach 10% at the end of the experiment. More specifically, in this experiment, the strain was concentrated at 10,000× *g* and was continuously concentrated while performing three repetitive centrifugations. The damage to the cell membrane of the strain during centrifugation could not be avoided. The low effective amount of the strain in the 5% inoculum system resulted in a slow increase in the oxidation efficiency, while the addition of fulvic acid to the 5% inoculum system exhibited high efficiency in the oxidation of Fe^2+^ and the system was completely oxidized after 96 h. The fulvic acid accelerated the electron transfer and provided the necessary energy for the strain. On top of that, the added calcium ions repaired the cell membrane and further improved the integrity of the bacterial cell membrane, whereas the Fe^2+^ oxidation was significantly accelerated under the action of the electron shuttle. The incorporation of fulvic acid and the increase in the inoculum of *A. ferrooxidans* had a promoting impact on the increase of the Fe^2+^ oxidation efficiency.

### 3.5. Kinetic Analysis under Different Inoculum Amount of A. ferrooxidans

**Figure 5:**
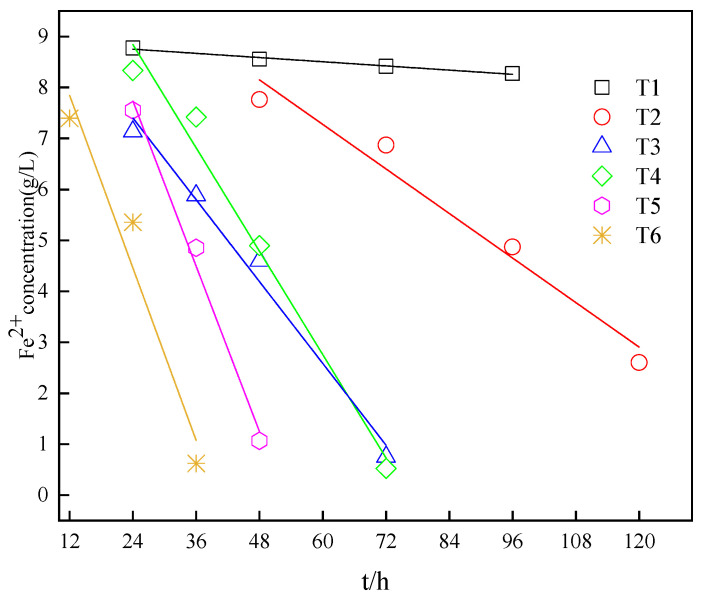
C-t curves (T1: 5% *A.f*; T2: 10% *A.f*; T3: 20% *A.f*; T4: 5% *A.f*, FA—0.2 g/L; T5: 10% *A.f*, FA—0.2 g/L; and T6: 20% *A.f*, FA—0.2 g/L).

The experiment was carried out without adding any nutrient solution. There was also no growth and reproduction, and only the activity of the bacteria was reflected. After the recovery of the bacterial activity, the oxidation rate was theoretically stable during the oxidation. The oxidation of Fe^2+^ by *A. ferrooxidans* under the present experimental conditions can be regarded as a first-order reaction since the C–t relationship is linear at different bacterial densities, as shown in Figure 5. The kinetic equation of Fe^2+^ oxidation and the reaction rate constants are presented in Table 1, where it can be observed that the reaction rate constants ranged from 0.007 to 0.282 h^−1^ with 0.2 g/L fulvic acid, and the highest oxidation rate was attained at 20% inoculum of *A. ferrooxidans*. A correlation coefficient of 0.977 can be seen when the *A. ferrooxidans* inoculum was 5%, with less correlation to its fitted linearity. While better fitting results have been reported by the other works in the literature, it should be underlined that *A. ferrooxidans* exhibited good stability after the recovery of the bacterial activity when the oxidation of Fe^2+^ was carried out. As can be observed from Figure 4, both the 20% *A. ferrooxidans* inoculum amount and the 5% fulvic acid added systems were completely oxidized after 96 h. The correlation coefficients of 0.980 and 0.972 from Table 1 showed a high correlation and rate constants of 0.134 and 0.169 g/L·h^−1^, respectively, with the latter having a higher oxidation rate over a longer period of time.

### 3.6. Impact of A. ferrooxidans Inoculum Amount on the TFe Precipitation Efficiency in Secondary Mineral Synthesis Systems

**Figure 6:**
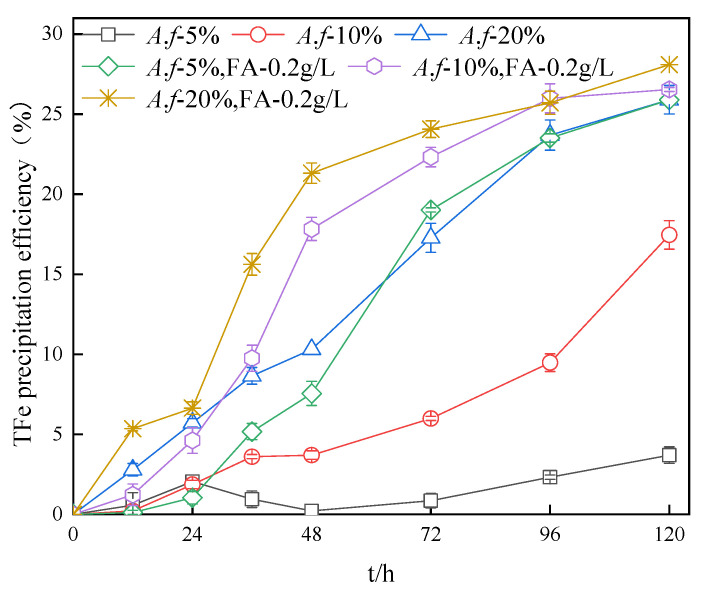
Impact of *A. ferrooxidans* inoculation amount on the TFe precipitation efficiency in systems.

*A. ferrooxidans* catalyzed the oxidation of Fe^2+^ to Fe^3+^, which was then hydrolyzed and mineralized. According to Figure 6, the increase in the inoculum amount contributes to the increase in the TFe precipitation efficiency with or without the addition of fulvic acid. On the one hand, the increase in the inoculum amount accelerated the oxidation of Fe^2+^ and enhanced the supply of Fe^3+^. On the other hand, the use of *A. ferrooxidans* as the central site of the crystalline nucleation promoted the formation and growth of minerals. This possibility was further supported by the fact that the efficiency of Fe^2+^ oxidation was higher after the dissolution of the extracted secondary minerals than before dissolution at the same time [33]. Compared with the experimental group without fulvic acid, a reduced amount of bacterial inoculation enhanced the promotion effect of fulvic acid. For example, after 96 h, the TFe precipitation efficiency was 23.7% with 20% inoculum amount and 25.7% with fulvic acid, which indicates an increase of 2%. Similarly, after 96 h, the TFe precipitation efficiency was 9.5% with a 5% inoculum amount and 26.0% with fulvic acid, which suggests an increase of 16.5%. It is noteworthy that the system with 20% inoculum and the system with a 5% inoculum and simultaneous addition of fulvic acid completely oxidized Fe^2+^ after 72 h, and the subsequent TFe precipitation efficiency was similar. Thus, it can be argued that there is some experimental equivalence between the effect of fulvic acid and inoculum amount on the promotion of Fe^3+^ hydrolysis.

### 3.7. XRD Analysis

**Figure 7:**
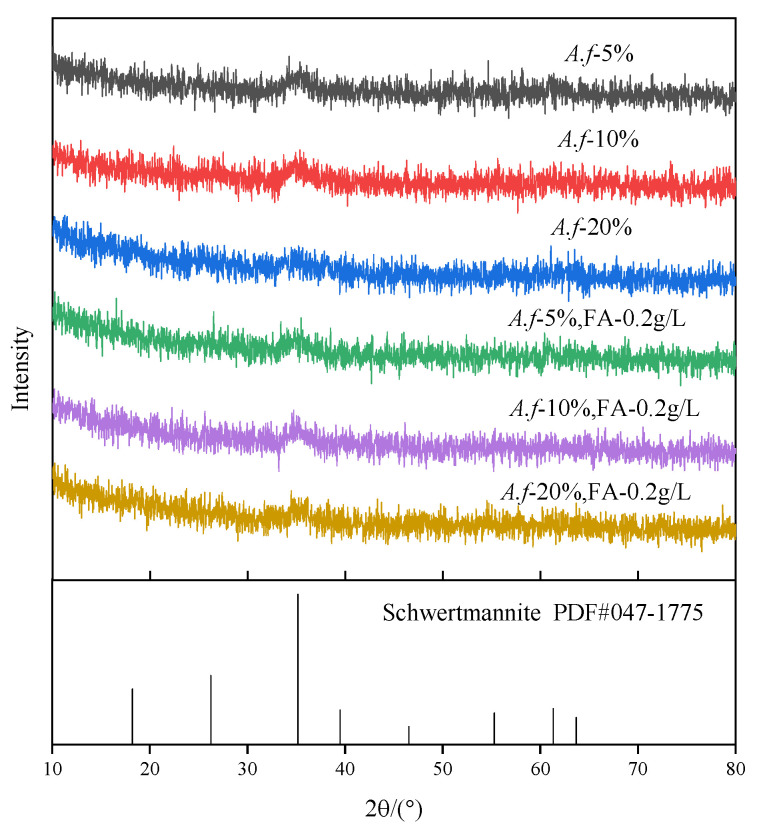
XRD atlas under different *A. ferrooxidans* inoculation amounts.

The precipitates obtained in the experiment were reddish brown hydroxyl sulfate secondary minerals, and the X-ray diffraction patterns are depicted in Figure 7. X-ray diffraction is considered one of the most effective means to distinguish crystalline minerals from amorphous minerals [34]. The crystalline minerals are capable of producing sharp X-ray diffraction phenomena, while the existence of K^+^, Na^+^, and NH_4_^+^ can induce the mineralization to form the crystalline minerals jarosite (K^+^, Na^+^, NH_4_^+^)Fe_3_(SO_4_)_2_(OH)_6_ [35]. Moreover, schwertmannite, Fe_8_O_8_(OH)_6_(SO_4_)_4_, is an amorphous mineral with eight characteristic broad peaks. As can be ascertained from Figure 7, the resulting secondary minerals were extremely poorly crystalline, with only one broad peak observed, and the spectrum was almost identical under the present experimental conditions.

### 3.8. FTIR Analysis

**Figure 8:**
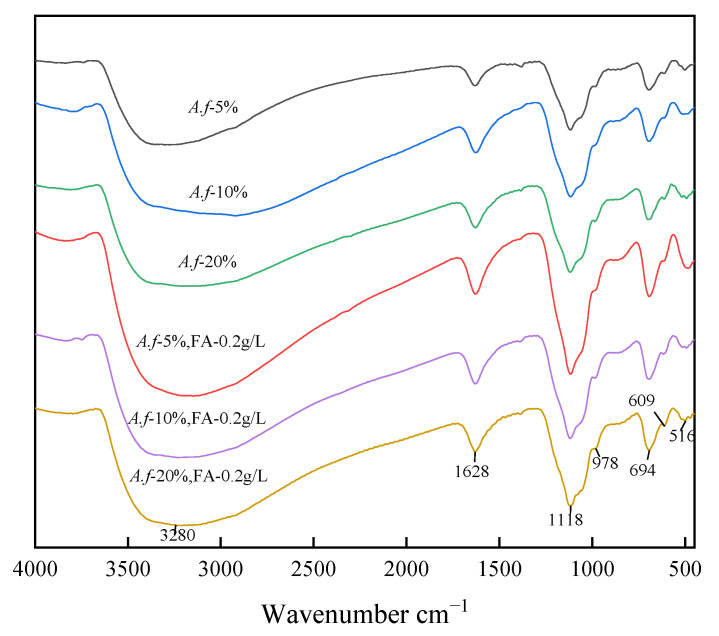
FTIR atlas under different *A. ferrooxidans* inoculation.

The FTIR spectra of the secondary minerals that were obtained under the experimental conditions are shown in Figure 8. In the spectrum, 3600–2500 cm^−1^ stands for the stretching vibration of OH, 1700–1500 cm^−1^ represents the deformation vibration of H-O-H, 1200–1100 cm^−1^ denotes the ν_3_ band of SO_4_^2−^, 1000–900 cm^−1^ refers to the ν_1_ band of SO_4_^2−^, and 710–690 cm^−1^ is the ν_4_ band of SO_4_^2−^ [36], which is the vibration peak of O-H...SO_4_^2−^. Furthermore, 610–600 cm^−1^ is the absorption peak of SO_4_^2−^ group vibration, and around 520–500 cm^−1^ appears as the vibration peak of the FeO_6_ octahedron [37]. The secondary minerals that were obtained by observing the spectrum do not contain hydronium jarosite, and each experimental group was obtained as pure schwertmannite.

## 4. Conclusions

1. When the concentration of the fulvic acid was less than 0.2 g/L, the enhancement of the concentration of fulvic acid could promote the activity of *A. ferrooxidans*. When the concentration of the fulvic acid was greater than 0.2 g/L, the activity of the bacteria gradually decreased as the fulvic acid concentration increased, indicating the manifestation of an inhibitory effect. The low concentration of fulvic acid also had a promoting effect on the precipitation efficiency of TFe in the system, while the high concentration of fulvic acid yielded an inhibiting effect on the precipitation efficiency of TFe.
2. The oxidation efficiency of *A. ferrooxidans* in the system with fulvic acid was higher than that of the system with the same inoculation amount at the same time. Interestingly, a reduced amount of the inoculum of *A. ferrooxidans* led to a more obvious oxidation effect. The oxidation activity of *A. ferrooxidans* in the system was in direct accordance with the first-order reaction equation. The increase in the *A. ferrooxidans* inoculation amount also accelerated the oxidation of Fe^2+^ and increased the precipitation efficiency of TFe.
3. The changes in the concentration of 0.2 g/L fulvic acid and the bacterial load did not affect the mineral phase and the functional groups of the secondary minerals in the system, and pure schwertmannite was obtained.

## Figures and Tables

**Table 1:** Kinetic equations and rate constants for Fe^2+^ oxidation.

Initial Conditions	Kinetic Equation	Correlation Coefficient R^2^	Rate Constants (g/L·h^−1^)
5% *A.f*	C = −0.007X + 8.916	0.977	0.007
10% *A.f*	C = −0.073X + 11.646	0.952	0.073
20% *A.f*	C = −0.134X + 10.613	0.980	0.134
5% *A.f*, FA—0.2 g/L	C = −0.169X + 12.897	0.972	0.169
10% *A.f*, FA—0.2 g/L	C = −0.270X + 14.218	0.981	0.270
20% *A.f*, FA—0.2 g/L	C = −0.282X + 11.231	0.900	0.282

## Data Availability

Not applicable.
